# Assessing the role of servicing in enhancing sanitation-related quality of life among container-based sanitation users

**DOI:** 10.1038/s44221-025-00508-6

**Published:** 2025-09-25

**Authors:** Benjamin Exton, Ana Casas, Amy Lewis, Simon Willcock, Beata Kupiec-Teahan, Dani J. Barrington, Fiona Anciano, Paul Hutchings, Andrew R. Bell, Mmeli Dube, Caroline Karani, Arturo Llaxacondor, Hellen López, Alesia D. Ofori, Joy N. Riungu, Kory C. Russel, Alison Parker

**Affiliations:** 1 Faculty of Engineering and Applied Sciences, Cranfield University, Cranfield, UK; 2 School of Environmental and Natural Sciences, Bangor University, Bangor, UK; 3 Net Zero and Resilient Farming, Rothamsted Research, Harpenden, UK; 4 Bangor Business School, Bangor University, Bangor, UK; 5 School of Population and Global Health, The University of Western Australia, Crawley, Western Australia Australia; 6 Department of Political Studies, University of the Western Cape, Cape Town, South Africa; 7 School of Business, Economics and IT, University West, Trollhättan, Sweden; 8 School of Civil Engineering, University of Leeds, Leeds, UK; 9 Department of Global Development, Cornell University, Ithaca, NY USA; 10 Meru University of Science and Technology, Meru, Kenya; 11 Sanima, Lima, Peru; 12 Department of Management Science, Pontificia Universidad Católica del Perú, Lima, Peru; 13 Department of Landscape Architecture and the Environmental Studies Program, University of Oregon, Eugene, OR USA

**Keywords:** Development studies, Environmental studies

## Abstract

Here we evaluate the servicing of container-based sanitation (CBS)—which includes the collection, replacement and cleaning of cartridges—and its influence on sanitation-related quality of life (using the SanQoL-5 index) in informal settlements across Kenya, Peru and South Africa. We (1) compared the incidence and severity of problems associated with CBS toilets against other sanitation types, (2) assessed the quality of CBS servicing across different regions and implementations and (3) evaluated the relationship between servicing issues and sanitation-related quality of life, utilizing high-frequency longitudinal smartphone survey data collected at various intervals over 1 year. Results revealed significantly fewer and less severe issues were recorded for CBS toilets than other toilet types, such as pit latrines, sewers and open drains. CBS servicing was consistently well regarded in all countries. Participants in Kenya highlighted particular satisfaction with the frequency of container replacement, whereas, in Peru, the cleanliness of replacement containers was highly regarded. SanQoL-5 scores decreased when CBS servicing issues were recorded, particularly in Kenya. This study underscores the potential of CBS as a sustainable sanitation solution in urban informal settlements, provided that high-quality servicing is maintained.

## Abstract (web summary)

A study showing high-quality container-based sanitation servicing improves the sanitation-related quality of life in informal settlements across Kenya, Peru and South Africa.

## Main

For over 1 billion people, sanitation in informal settlements remains a critical issue that directly impacts public health, environmental sustainability and their overall quality of life^1^. Informal settlements are characterized by high population density, inadequate infrastructure, limited financial resources and unclear land ownership^2–4^, which make it difficult to implement effective sanitation solutions^5^. Tackling global sanitation is a crucial element of the United Nations’ Sustainable Development Goal 6 (SDG 6), which aims to ensure the availability and sustainable management of water and sanitation for all by the year 2030 ref. ^6^. While progress has been made towards SDG 6, globally the goals are not currently on track to be met^7–9^. To ensure the availability and sustainability of sanitation for all, improving the quality and affordability of toilets in informal settlements is essential—especially as rapid urbanization drives a growing population into urban informal settlements^10,11^.

Several toilet types are commonplace in informal settlements, each with distinct advantages and limitations, influenced by cost, location and waste disposal methods^12–14^. Piped sewer systems enable efficient waste removal but are hard to implement in lower-income regions due to high installation and maintenance costs^15,16^. Furthermore, the challenges of unclear land ownership and the precarious legal status of informal settlements render it impractical to implement permanent infrastructure, such as piped sewer systems, in many locations^17^. By contrast, less permanent solutions such as pit latrines, open drains and hanging toilets are common because of their low cost and simplicity^15,18^. However, these solutions pose substantial challenges, including difficulties in emptying, health risks from groundwater contamination—particularly in flood-prone areas—and direct expose of users to faecal waste^19,20^. Composting toilets offer cobenefits for communities but have higher costs and demand proper maintenance^21,22^. Alternative temporary sanitation solutions that contain waste and facilitate proper disposal, such as informal bucket systems, offer improvement, but the manual handling of waste still presents serious health risks^23,24^.

Container-based sanitation (CBS)^25^ has gained prominence as a viable off-grid solution for managing human waste in rapidly urbanizing and resource-constrained environments^26,27^. CBS operates through the containment of urine and faecal matter within sealable containers, which are regularly collected and transported to centralized treatment facilities, often under a subscription-based model^2,28,29^. This collection is usually referred to as ‘servicing’. CBS toilets have various designs—for example, the separation faeces and urine—while others collect both together for offsite treatment. A cover material such as sawdust or ash is often added to help reduce odours. CBS systems are designed to be small, adaptable and/or transportable, making them particularly suitable for densely populated urban areas where land, resources and security of tenancy are scarce^30^.

CBS is not without its challenges as there are difficulties in implementation, ongoing servicing and maintenance, in addition to complexities in scaling up to meet demand^2,10,27,31^. The effectiveness of CBS depends on the reliability and efficiency of the entire service chain, which includes the condition and cleanliness of containers, collection and transportation of containers, as well as treatment and disposal of waste. Each of these stages must be managed by a CBS provider—whether a social enterprise, non-governmental organization (NGO) or municipal body—to ensure the system’s sustainability. Effective servicing of CBS systems is essential to the schemes success and improving sanitation-related quality of life in low-income informal settlements. The sanitation-related quality of life (SanQoL-5) index, based on the capability approach, assesses the impact of sanitation on well-being beyond traditional health outcomes by focusing on five attributes: disgust, privacy, safety, health and shame^32^. This framework captures how sanitation influences dignity, security and social status to capture what people value about sanitation beyond traditional health impact justifications.

The primary aim of this study is to evaluate users’ satisfaction with the servicing of CBS toilets and the relationship to overall sanitation satisfaction. It is hypothesized that (1) CBS users experience fewer and less severe problems with their toilets compared with non-CBS users, (2) servicing of CBS toilets is reported on positively by users and (3) high-quality servicing of CBS toilets leads to improved sanitation-related quality of life.

In this study, high-frequency longitudinal smartphone surveys were conducted over 1 year to address these hypotheses. Approximately 100 participants from three informal settlements in Kenya (Mukuru Kwa Reuben, Nairobi), Peru (Pamplona Alta, Lima) and South Africa (BM Section of Khayelitsha, Cape Town) were surveyed with an even split between CBS and non-CBS users and also between adult males and females. The participants were compensated for responding to surveys with phone ownership, data and talk time—further breakdown is given in ref. ^33^. In Kenya and Peru CBS schemes are provided through a subscription model by social enterprises with grants and donor support (covering approximately 80% of service costs (ref. ^34^ and F.A., M.D., A.M. & A.O., manuscript in preparation)), whereas in South Africa CBS is provided by the local municipality for free and at a much larger scale. Several survey modules were administered at different frequencies (weekly, monthly, quarterly or once—depending on question type^33^) but of highest relevance in this study are the data gathered with weekly well-being and sanitation questionnaires^33^. Smartphone survey data were systematically gathered and anonymized, followed by an extensive cleaning process to ensure accuracy and reliability as detailed in ref. ^33^. Both CBS users and non-CBS participants recorded problems with their toilets, self-assessing severity using categorical options (minor, moderate and serious), and CBS users were asked about the quality of CBS servicing and ratings of their sanitation well-being, later used for calculation of the SanQoL-5 index^32^. Responses to servicing and SanQoL-5 questions were normalized to give an overall score for each metric.

## Results

### Problems reported with different toilet systems

After cleaning and filtering data, 7,121 responses to the two relevant questions on problems with their toilet were retained, a total of 53.9% of which were from Kenya, 23.6% from Peru and 22.5% from South Africa (Supplementary Appendices A-1 and A-2). Responses were further categorized as CBS users (52.9% of responses) and non-CBS users (47.1%), which remained approximately even in each country. The mean percentage engagements were 81.5% in Kenya, 64.5% in Peru and 50.5% in South Africa from 108, 96 and 98 participants, respectively, for these two weekly questions over approximately 52 weeks. The primary toilet type for non-CBS users varied by country. In Kenya, ‘flush to sewer’ was the most common (69.9% responses), whereas responses were more diverse in Peru—flush to pit (33.9%), pit latrine with slab (13.7%), flush to sewer (11.8%) and open pit latrine (10.5%)—and South Africa, that is, sewer (37.8%), flush to unknown destination (14.9%) and flush to open drain (12%).

**Fig. 1:**
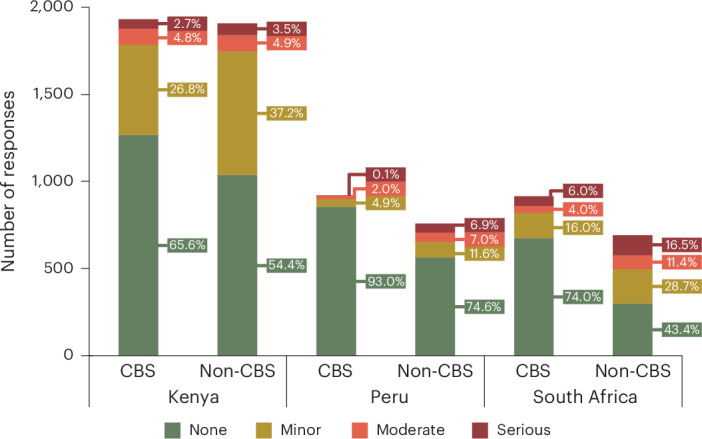
Problems reported with different toilet systems. Recorded problems with participants’ toilets by CBS users and non-CBS users per country in weekly sanitation questions. The colours denote the severity of the reported issues.

Every week, participants were asked ‘Did you have any problems with the toilet itself this week?’ and to self-report the severity/impact of that problem (‘minor’, ‘moderate’ and ‘serious’). The majority of responses from CBS users indicated no problem (65.6% of CBS-user responses in Kenya, 93.0% in Peru and 74.0% in South Africa), whereas non-CBS participants recorded significantly more problems (54.0% of non-CBS responded ‘no problem’ in Kenya, 74.6% in Peru and 43.4% in South Africa; *P* < 0.001 comparing non-CBS and CBS users in each country, chi-squared test) (Fig. 1). Calculating the problem rate per participant (number of problems reported divided by total number of responses), we can use a generalized linear model with a binomial distribution and logit link function to take into account these country level effects, and this shows CBS users have a lower problem rate than non-CBS (coefficient of −0.367; 95% confidence interval −0.627 to −0.113; *P* < 0.01). There was no significant interaction for problem rate between country and CBS/non-CBS (*P* > 0.05).

The severity of recorded problems was primarily confined to ‘minor’ but varied by country and by toilet type (Fig. 1). Not only did CBS users report fewer problems, but they also reported a significantly lower severity of problems in each country (18.8% of responses were ‘minor’, 3.9% ‘moderate’ and 2.9% ‘serious’) compared with non-CBS users (29.7% ‘minor’, 6.7% ‘moderate’ and 6.9% ‘serious’; *P* < 0.001 for each individual country (tested separately), chi-squared tests). Comparing responses between the different countries, CBS users in Kenya had a significantly higher proportion of ‘minor’ problems (26.8% responses) than South Africa (16.0%; *P* < 0.001) and Peru (4.9%; *P* < 0.0001). However, CBS users in South Africa had a significantly higher proportion of ‘serious’ problems (6.0% responses) than Kenya (2.7%; *P* < 0.0001) or Peru (0.1%; *P* < 0.0001). We observed similar patterns in comparisons between countries of non-CBS users.

Participants were also asked about the specific problems, including bad smell, toilet needed emptying, toilet needed repairs and ‘other’. The specific type of problem with a CBS-user’s toilet were broadly similar between countries (Supplementary Appendix A-3). Bad smell was consistently the highest proportion of recorded problems by CBS users (39.0–56.6% of responses by country). In Kenya and South Africa, this was followed by the toilet needing emptying (30.1 and 26.1% of responses, respectively) and repairs needed (21.4 and 23.7%), whereas in Peru, repairs required were fractionally more widely reported (15.8%) than the toilet needing emptying (14.5%). The most commonly reported problem under ‘other’ broadly related to the toilet being broken, with other commonly reported problems being insufficient water (for flushing and hand washing), poor cleanliness, insufficient hand washing facilities (soap and water) and issues with accessibility (the toilet closing early and opening late, as well as the distance of travel to the toilet).

### Satisfaction with CBS servicing

**Fig. 2:**
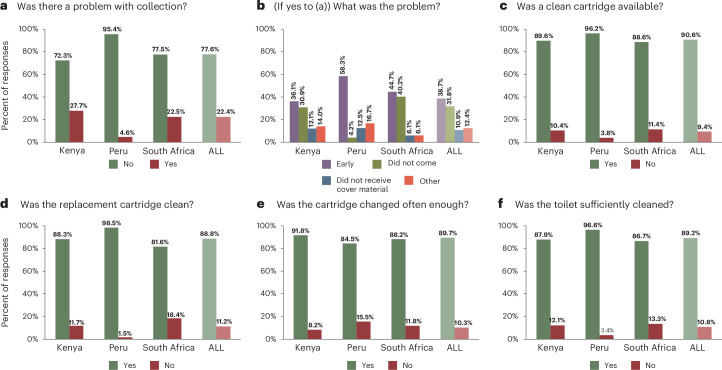
Satisfaction with CBS servicing. **a**–**f**, Responses to servicing questions in Table 2a: problem with collection (**a**), what type of problem (collector early, collector did not come, cover material (sawdust) not received, do not know and/or other) (**b**), clean cartridge availability (**c**), cleanliness of replacement cartridge (**d**), sufficiency of cartridge changes (**e**) and sufficiency of toilet cleaning (**f**). The percentage responses were calculated per country, with overall average (‘ALL’, faded colour).

CBS users were also asked weekly questions about the quality of servicing of their CBS toilets (Fig. 2). Data were filtered to retain only responses from CBS users that completed weekly sanitation and well-being questionnaires resulting in 2,834 valid responses for the six relevant questions in Fig. 2, of which 60.9% were from Kenya, 18.5% from Peru and 20.6% from South Africa (Supplementary Appendix B-1). The mean percentage engagements were 63.1% in Kenya, 25.6% in Peru and 22.2% in South Africa from 59, 40 and 67 participants, respectively, for these six weekly questions over approximately 52 weeks.

The overwhelming proportion of responses to servicing questions were positive (72.3–98.5% positive responses by question) (Fig. 2, green bars), reflecting a general satisfaction with the servicing of CBS toilets. As a result, it is harder to identify those questions where positive responses were disproportionately high or low compared with negative responses; therefore, the positive-to-negative ratio was calculated for each servicing question (Supplementary Appendix B-2). Notably, Peru had a much higher proportion of positive (95.4–98.5%)-to-negative (1.5–4.6%) responses (that is, 20.8–64.5× more positive-to-negative responses for each servicing question) than either Kenya or South Africa (2.6–11.3× and 3.4–7.8×, respectively), except for the frequency of container collection question which was poorer (5.5×) (Fig. 2e). Participants in Kenya recorded the frequency of bucket collection most positively of servicing questions (11.3× more positive-to-negative responses) (Fig. 2e) but a comparatively larger proportion of overall problems with their toilet in any given week (2.6×) (Fig. 2a) (Supplementary Appendix B-2). Peruvian participants were overwhelmingly positive about the cleanliness of their replacement cartridges (64.5× more positive-to-negative responses) (Fig. 2d), whereas South Africa had the lowest average ratio of positive-to-negative responses (an average of 5.9× more positive-to-negative responses), with the availability of clean cartridges (7.8×) (Fig. 2d) and the frequency of bucket collection (7.5×) (Fig. 2) recorded most positively.

If participants responded that they had a problem with collection that week (Fig. 2a), they were asked what that problem was (Fig. 2b). In all countries, the highest proportion of problems were because the collector came before containers had been placed outside (38.7% of all responses). This was especially true in Peru (58.3% of country’s responses). By contrast, Kenya and South Africa had a similar number of responses that the collector did not come (30.9% and 40.2%, respectively) compared with that the collector came before containers had been put outside (36.1% and 44.7%, respectively).

### The relationship between CBS servicing and sanitation-related quality of life

After combining data from servicing and SanQoL-5 questions and filtering to retain only those participants and the weeks where both sets of questions were answered, 1,914 responses from 126 participants with CBS were retained: 49 from Kenya, 35 from Peru and 42 from South Africa (Supplementary Appendix C-1). The average number of responses from each participant varied by country (Supplementary Appendices C-4 and C-5). Kenya had the highest median number of responses per participant (31 responses per participant) compared with Peru (7) and South Africa (10) in this filtered dataset.

**Fig. 3:**
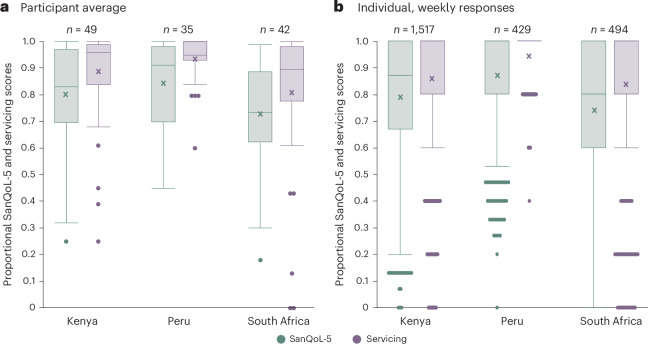
The relationship between CBS servicing and sanitation-related quality of life. **a**,**b**, A summary of participant-average scores (**a**) and individual, weekly scores per participant (**b**) for sanitation-related quality of life (teal) and CBS servicing (purple). ‘Participant-average’ scores are mean-averaged for each participant. ‘Individual, weekly’ scores are all individual datapoints. Individual participant responses better capture outlier values (for example, weeks where there was a problem) but are skewed by participants with higher percent engagement which average participant responses mitigate. The boxes are the interquartile range with median as middle line and mean as a cross, and the whiskers are the range up to 1.5× the interquartile range with dots outside this representing outliers. *n*, number of data points used in each box plot.

The median participant-average servicing scores (0.895–0.960) were higher than median SanQoL-5 scores (0.735–0.910 by country) (Fig. 3a and Supplementary Appendix C-6), though it is important to note that the 0–1 scales for these metrics reflect different constructs. Participant-average servicing scores in Kenya and Peru also had a smaller interquartile range (0.150 and 0.070, respectively) than SanQoL-5 scores (0.275 and 0.280, respectively), compared with South Africa which had a similarly large spread of SanQoL-5 and servicing scores (interquartile range of 0.205 and 0.265, respectively)—although this is probably an artifact of higher mean average and an upper-bound constraint. Therefore, in general, participants had wider opinions on their sanitation-related quality of life than on the servicing of their CBS toilets and, on average, had a higher opinion of their CBS toilets servicing than their sanitation well-being.

When evaluating individual weekly responses of participants to SanQoL-5 and servicing questions, the median scores for SanQoL-5 (0.870 in Kenya, 1.000 in Peru and 0.800 in South Africa) and servicing questions (1.000 for all countries) are higher than participant-average scores (Fig. 3b and Supplementary Appendix C-7). Moreover, the lower bound of the interquartile range remains a ‘perfect’ servicing score of 1.000 in Peru. The mean averages of both participant-average scores and individual weekly scores are lower than their respective medians and is probably caused by infrequent—but nevertheless present—low weekly scores influencing mean weekly scores—because, for example, there was a problem with the participants toilet that week. The outliers in box plots (Fig. 3b) accounted for 2.3% and 12.8% of responses for SanQoL-5 and servicing scores of individual weekly participant scores, respectively.

There is a substantial difference in the participant-average servicing scores between different CBS implementations—social enterprise (median servicing score of 0.960 in Kenya and 0.950 in Peru) compared with municipality (0.895 in South Africa)-run services (Fig. 3a)—with a significant difference between Peru and South Africa (*P* = 0.0142, pairwise Wilcoxon post hoc test following Kruskal–Wallis). The differences between CBS implementations are further reflected in SanQoL-5 scores where South Africa has a significantly lower participant-average score (median of 0.735) compared with Kenya (0.830; *P* = 0.0182) and Peru (0.910; *P* = 0.0025).

### The influence of CBS servicing on participants’ sanitation-related quality of life

**Fig. 4:**
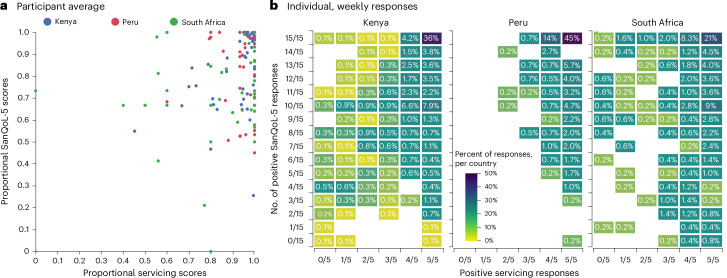
The influence of CBS servicing on participants’ sanitation-related quality of life. **a**,**b**, The relationship between SanQoL-5 and CBS servicing scores for mean (**a**) and individual (**b**) scores per participant per country surveyed: Kenya (blue), Peru (red) and South Africa (green). The mean scores are a continuous dataset and reflective of calculated values, whereas individual weekly scores are ordinal data.

Thus far, the analysis has focussed on a detailed understanding of the responses to SanQoL-5 and servicing questions separately; however, it is also important to evaluate the relationship between these two variables. Plotting participants’ servicing scores against the corresponding SanQoL-5 scores reinforces previous observations that participants were broadly highly satisfied with the servicing of their CBS toilets and had a moderate—but more varied—sanitation-related quality of life (Fig. 4). Because of the robust servicing of CBS toilets in these communities, it was difficult to infer any strong relationship between servicing quality and sanitation-related quality of life (Supplementary Appendix C-2). Consequently, the positive relationship between factors was not as strong as hypothesized but nevertheless highlighted the broad satisfaction with CBS servicing and sanitation-related quality of life of CBS users and is supported by other studies^35^. However, when participants reported issues with their CBS toilet servicing, there was a noticeable decline in the average SanQoL-5 score, particularly in Kenya (Fig. 4b). For instance, when participants responded negatively to at least one servicing-related question (servicing score ≤0.8 in individual weekly scores), the mean SanQoL-5 score in Kenya decreased by 16.1% compared with the overall mean and by 7.4% in South Africa, with a negligible change observed in Peruvian mean SanQoL-5 score. When two servicing questions were answered negatively (servicing score ≤0.6), the mean SanQoL-5 score in Kenya decreased by 33.0% compared with the overall mean, while smaller but nevertheless notable decreases were observed in Peru (7.1% decrease) and South Africa (12.3% decrease). Therefore, while overall satisfaction with CBS servicing may not show a strong correlation with aggregated SanQoL-5 metrics, specific negative experiences with servicing can impact participants sanitation-related quality of life, particularly in Kenya.

## Discussion

Using the data presented in this study, it is challenging to draw definitive conclusions about the influence of CBS toilet servicing on sanitation-related quality of life. Responses to CBS servicing questions were positive (average 87.2% positive compared with 12.8% negative responses) indicating a general satisfaction with CBS servicing in all the surveyed countries resulting in no strong linear trend between SanQoL-5 and servicing scores as hypothesized (Fig. 4). While there was no strong correlation, as hypothesized (Supplementary Appendix C-2), there are indications of links between sanitation-related quality of life and better servicing of CBS toilets—such as the mean SanQoL-5 score decreasing when one or more servicing questions were answered negatively. To better elucidate these relationships SanQoL-5 and servicing scores were looked at in two ways: participant-average and individual weekly scores. Each approach offered valuable insights but had their own limitations. Participant-average scores had the benefit of not skewing the overall dataset towards participants who responded more frequently to the smartphone surveys (Supplementary Appendix C-4). Whereas individual weekly scores highlighted weeks where participants responded more negatively to SanQoL-5 and/or servicing questions, indicating when there was a problem and providing valuable insights into shocks and how a decrease in the score of one variable influenced the other. Moreover, there was no notable change in either SanQoL-5 or servicing scores, indicating that time has no substantial influence over participant-average nor individual weekly scores (Supplementary Appendix C-3, albeit with a low *R*^2^ of linear models).

### Implementations of CBS in different informal settlements

While other studies have evaluated different CBS implementations^35,36^, this study, for the first time, monitored CBS satisfaction alongside the SanQoL-5 index at high-frequency. The differences between CBS implementations may also explain observed differences in reported problems with toilets as well as the servicing and sanitation-related quality of life for CBS users between countries. For example, the higher servicing and SanQoL-5 scores in Kenya and Peru may indicate that the social enterprise run schemes have better servicing and sanitation-related quality of life than municipal run scheme in South Africa. While this finding is supported by similar studies^24,37,38^, the scale of implementations and societal attitudes to sanitation must also be considered^10,34,39^.

Each of the study locations has a unique socioeconomic environment and societal attitudes towards sanitation, as well as a different implementation of CBS. In South Africa, there is a higher demand and expectation for adequate sanitation provision^10^. As a result, the City of Cape Town provides CBS services for free to approximately 30,000 people (ref. ^10^ and M.D., F.A., D.B. & A.O., manuscript in preparation), whereas, in Lima (Peru), Sanima serves around 7,500 people^34^. While free, the service in Cape Town is often found to be undignified, with broken or inadequate servicing and unable to keep up with rapid urbanization^10,40^. In comparison, NGO/donor backed social enterprises in Kenya and Peru charge users less than the cost of operation and are supported by grant funding and donations (accounting for approximately 80% of the service cost) (ref. ^34^ and F.A., M.D., A.M. & A.O., manuscript in preparation). Findings from this study showed that the social enterprise schemes in Kenya in Peru had fewer and less severe problems and aligns with similar studies^24,37,38^; however, no direct causal relationship was established here, and the scale of operation is much greater in South Africa (local municipality) than Keyna or Peru (social enterprises), which puts additional strain on the sanitation regime.

However, numerous other factors can influence users’ satisfaction with CBS servicing quality, such as the governance structures and policies surrounding sanitation in each country. South Africa has a ‘monolithic’ sanitation system where there is a clear body responsible for sanitation and a greater expectation by residents^39,41^. Conversely, Peru is categorized as a ‘fragmented’ system, where several service regimes are responsible for aspects of sanitation^42,43^, and a ‘splintered’ sanitation governance in Kenya, where the degree of fragmentation across state departments is utterly misaligned with absolute lack of interoperability^44^. As a result, in Kenya and Peru, NGO backed social enterprises try to fill in gaps and support improved sanitation programs in urban, informal settlements^31,45,46^; however, in Kenya, a vacuum emerged that landlords and cartels stepped into providing unsafely managed sanitation^39,47,48^, which reflects the weaker moral economy of sanitation in Kenya (F.A., M.D., A.M. & A.O., manuscript in preparation). By contrast, Peru has seen the implementation of several trial sanitation schemes^49,50^. Yet, the multiple services in Kenya and Peru are operated amid a fragmented sectoral regime and weak political drive. Sanitation provision for the urban poor and marginalized in Peru remains a low priority, hindered by insufficient financing, the ‘state capture’ of sanitation services, widespread corruption and a ‘muddling through’ management approach^51^, despite commitments and aspirations to achieve adequate sanitation^52^.

Following apartheid in South Africa, there has been a strong social contract and activism to ensure the provision of sanitation in informal settlements^41,53–55^. In South Africa, where there is a history of social activism and a strong social contract regarding the right to sanitation, residents have higher expectations and are demanding of sanitation standards^56^. These higher expectations may manifest as lower satisfaction with servicing and sanitation-related quality of life, as presented in this study. Whereas in Kenya, despite the mandate in their constitution^57^, the state is much less involved, and residents of informal settlements view sanitation as a private affair with a much lower societal drive to demand improvement^39,58–60^. In Kenya, there is a more widespread acceptance of the status quo, which is probably reflected in higher Servicing and SanQoL-5 scores presented in this study. The difference in societal attitudes towards sanitation, despite similar legislative mandates, probably influences residents’ perceptions and satisfaction with the services provided. These differing attitudes highlight the broader global disparities in perceptions and expectations regarding the human right to dignified sanitation within the established international frameworks^61,62^.

To broaden out these findings, in Kampala (Uganda), rapid population growth in the mid-1990s strained government sanitation services which deteriorated, prompting a shift to private sector involvement^63^. Examining this example, Tukahirwa et al. (2010) found that effective sanitation required collaboration between government, NGOs and private partners^64^—though some financial and political challenges persisted^63^. These challenges mirrors findings in Kenya, Peru and South Africa in this study, where issues with CBS servicing also impacted sanitation-related quality of life, highlighting the need for strong, genuine cooperation between state, private, NGO and community partners to maintain effective sanitation.

Taken together, the reasons for servicing quality and the influences on sanitation-related quality of life are complex and not fully explained by the data presented in this study and may be reflective of the wider sanitation systems and attitudes in each country. For example, while Kenya has higher overall mean scores (*x̄* = 86% servicing score and 79% SanQoL-5 score) compared with South Africa (*x̄* = 84% and 74%, respectively), there is a more pronounced decrease in SanQoL-5 scores with diminishing quality of servicing of CBS toilets in Kenya. When at least two servicing questions were answered negatively, there was a 33.0% decrease in mean SanQoL-5 score in Kenya compared with a 12.3% decrease in South Africa. The resilience in South African SanQoL-5 scores may reflect its more structured sanitation governance, where a designated authority provides CBS services free of charge to informal settlement residents coupled with a stronger societal sense of empowerment for service improvements. By contrast, Kenya’s CBS market is dominated by social enterprises operating in an environment shaped by cartels and limited state support, potentially exacerbating the consequences of servicing issues^47,65^.

### Is the servicing of CBS toilets adequate?

In weekly questions to all participants, CBS users reported significantly fewer problems with their toilets, with a 27.8% higher prevalence of issues among non-CBS users. Moreover, the problems encountered by CBS users were generally minor in severity, contrasting with the more frequent occurrence of moderate and serious issues reported by non-CBS users (Fig. 1). Evaluating specific problems of CBS users revealed smell to be the most common issue—similarly reflected in literature^66^—whereas the need for repairs or emptying were less prevalent. Smell is a substantial stigma in satisfaction with toilet cleanliness and toilets are commonly avoided if they smell^67^, disincentivizing CBS use if there are poor odours. Bad smells from CBS toilets can arise from not using sufficient dry cover material (as a water-free ‘flushing’ alternative)^27,68,69^ but can also be a consequence of poorly maintained CBS systems^10^, emphasizing the need for proper maintenance and sufficient cover material. From data collected in this study, not having sufficient cover material, in CBS systems that require it, remained uncommon (only 6.5% of reported toilet problems) (Fig. 2b), suggesting insufficient maintenance of CBS systems could be responsible for poor odours.

Overall, CBS users expressed high satisfaction with the servicing of their toilets, with an average of 13.6× as many positive-to-negative responses to servicing questions across all surveyed countries. While there was no strong correlation between servicing satisfaction and SanQoL-5 scores (*R*^2^ always ≤0.3313) (Supplementary Appendix C-2), poorer servicing did correlate with a decrease in SanQoL-5, particularly in Kenya. These findings suggest that while overall satisfaction with CBS servicing is generally high, the quality and consistency of servicing can have a big impact on perceived sanitation-related quality of life, especially in contexts where sanitation infrastructure and public health are less robust. This is consistent with existing literature that highlights the importance of reliable and consistent sanitation services in improving quality of life in low-income and informal settlements^70^.

The efficacy of CBS as an affordable sanitation system in urban informal settlements depends on the reliability and quality of its servicing. The analysis presented here indicates that the servicing of CBS systems is highly regarded as positive by users. These findings suggest that CBS can serve as a viable alternative to permanent improved sanitation solutions (such as flush toilets connected to the main sewer network) in communities where it is implemented. However, CBS should still be seen as a temporary measure on the way to a permanent and integrated improved sanitation solution.

### Smartphone surveys and limitations

The use of smartphone surveys has gained increasing interest in recent years over household surveys, especially in developing countries^71–74^. These surveys offer advantages, such as for research conducted in hard-to-reach areas, the ability to reach a larger number of participants than traditional in-person interviews or questionnaires and reducing administrative burdens associated with paper-based methods, such as the risk of losing printed surveys. However, smartphone surveys are constrained by technological limitations, such as availability of sufficiently powerful smartphones capable of running survey applications without crashing or the uninstallation of apps with limited drive space^75^.

Participant engagement, the proportion of weeks each participant responded to well-being and sanitation questions, also presented a challenge in this study. A large sample size (~100 participants per location, split approximately in half by CBS and non-CBS users) was adopted to mitigate these influences but the rate of non-response can still present challenges when evaluating subsets of the dataset. The median engagement percentage of participants was significantly higher in Kenya (median of 59.6%) than Peru (13.5%; *P* < 0.0001, pairwise Wilcoxon post hoc test following Kruskal–Wallis) or South Africa (19.2%; *P* < 0.0001). The median average presents a starker difference between countries than the mean, which was influenced by the small number of participants with abnormally high response rates (Supplementary Appendices C-4 and C-5). In Peru 45.7% of participants and 28.6% in South Africa had ≤5 valid weekly responses for both SanQoL-5 and servicing questions. For this reason, descriptive percentages have been used where possible, rather than absolute values, to account for imbalances in the studies dataset. High rates of participant non-response, as seen in this and similar longitudinal studies, further complicate the use of smartphone surveys but is not a unique challenge to smartphone surveys (refs. ^73,74^ and A.C. et al., manuscript in preparation). A specific challenge influencing response rate was the difference in reward mechanisms, varying rates of smartphone ownership and socioeconomic factors between countries. For instance, in South Africa, the compensation provided to participants had to be increased mid-survey to mitigate high attrition rates, highlighting the difficulties in maintaining participant engagement in these settings^75^. Furthermore, attrition was compounded by the need to filter out poor-quality data or when combining datasets from different modules of questions for comparative analysis^33^, such as was observed here.

A further limitation of this study was the lack of explicit validation of the SanQoL-5 index within the specific contexts of the three settlements studied at the time (SanQoL-5 has since been validated in Kenya^76,77^). However, the core attributes of SanQoL-5—disgust, health, shame, safety and privacy—are widely recognized as critical sanitation concerns across diverse settings. The longitudinal design, with weekly measures and participant check-ins, provides a form of practical validation, while the use of a standard weighting system promotes comparability. Given the absence of any globally validated measure of sanitation-related quality of life, the SanQoL-5 index remains the most appropriate tool available for this research.

## Conclusion

Sustainable sanitation in informal settlements is a global challenge and is a key pillar of the United Nations SDG 6. CBS is a promising technology that reduces faecal contact compared to other low-cost, non-permanent alternatives (improving sanitation for users) and risk of environmental contamination (affecting public health), offering flexibility and reduced infrastructure costs through an affordable subscription model.

Using longitudinal smartphone survey data from users of different toilet types in informal settlements in Kenya, Peru and South Africa; we evaluated the recorded problems with different toilets, the servicing quality of CBS toilets in different implementations and the relationship of CBS servicing with participants’ SanQoL-5 metrics. There were significantly fewer problems with CBS users toilets compared to other toilet types and, when they did occur, problems were of a lower severity. Moreover, the servicing of CBS systems was consistently highly regarded by users with higher-quality servicing having links to improved sanitation-related quality of life. These findings not only underscore the importance of good maintenance and servicing of CBS systems but also highlight the successful implementation of this transformative technology.

## Methods

### Study locations and CBS implementations

In this study, we examine off-grid sanitation in informal settlements across three countries that, while exhibiting distinct implementations of CBS systems, share comparable socioeconomic conditions and challenges. The three informal settlements are in (1) Nairobi, Kenya; (2) Lima, Peru; and (3) Cape Town, South Africa^29^ (Table 1). These settlements were selected from six sites where CBS was being implemented globally to encompass both municipal provision and social enterprise models (supported by grant funding)^78^ and to enable comparisons between different schemes, their implementations and effectiveness.

**Table 1:** Overview of the informal settlements in this study

Settlement	Location	CBS operator	Container collection
Mukuru Kwa Reuben	Nairobi, Kenya	Social enterprise with grant funding	From home/toilet
Pamplona Alta	Lima, Peru	Social enterprise with grant funding	From collection point
BM Section, part of Khayelitsha settlement	Cape Town, South Africa	State	From collection point^a^

^a^Some CBS users in South Africa have ‘pullers’ who collect cartridges from users’ plot and transport them to a collection point; however, the service is irregular and unreliable.

### Survey methods and data cleaning

Smartphone survey data was gathered through a comprehensive and systematic approach as detailed in ref. ^33^. The surveys were conducted using ODK software^79^ via the Data Exchange app^80^ over the course of a year. Data collected included demographic information, well-being, sanitation, income, infrastructural service use and socioeconomic variables. These modules of questions were asked at different intervals, either weekly, monthly, quarterly or once^33^. Approximately 100 participants were surveyed in each country, half were CBS users and half non-CBS users with a range of different demographics (such as age and gender). Local teams in each country were involved in identifying 150 CBS users covering a balanced geographic spread over the study sites; from this, 50 households were randomly selected stratified by gender; each person named three demographically similar households nearby of which one was randomly selected, matching respondent gender—a full description of participant selection is given in ref. ^33^.

The collected data underwent a rigorous cleaning process to ensure its accuracy and integrity for subsequent analyses^33^. Initial steps included the removal of duplicate entries and records with excessive missing data. Subsequent cleaning involved the standardization of categorical variables and logical checks to identify contradictory responses. The cleaned dataset was subjected to a series of quality control measures including random sampling and cross-validation, to verify the integrity and reliability of the data. Furthermore, the data were anonymized to ensure participant confidentiality and comply with ethical standards. Ethical approval was obtained in all three countries.

### Data analysis

The datasets of particular relevance in this study were derived from questions focused on well-being and sanitation. Further data filtering was required, for example, to only capture respondents who, in any given week, answered the relevant questions on both well-being and sanitation. CBS users and non-CBS were defined on the basis of their categorization at the start of the survey.

### Type and severity of problems with different toilet systems in informal settlements

Each week, participants were asked ‘Did you have any problems with the toilet itself this week?’ with the option to select ‘none’, ‘minor’, ‘moderate’, ‘severe’ or ‘unknown’. Responses of ‘unknown’ were minimal for all toilet types (0.6–2.2% for CBS users and 1.8–5.6% for non-CBS for each country). Responses of ‘minor’, ‘moderate’ and ‘severe’ were grouped as responses indicating a problem, with ‘none’ categorized as no problem with their toilet that week. Respondents who selected that they had a problem were then asked ‘what kind of problem(s)’ as a multiple-choice question. Possible answers were: ‘smelled bad’, ‘needs emptying’, ‘needs repairs’, ‘other’ and ‘don’t know’. For responses of ‘other’, participants could complete a free-text answer.

### The servicing of CBS toilets and its influence on sanitation-related quality of life

Participants who were identified as CBS users were asked a range of yes/no questions about the servicing of their CBS toilet (Table 2a). Answers to these five questions were categorized as positive or negative, then given the value 1 or 0, respectively. Participants who reported a problem with their CBS toilet (Table 2a(i)) were also asked ‘What problem did you experience?’. Additional questions about toilet cleanliness (Table 2) (for example, ‘How often was the toilet cleaned this week?’ and ‘How long did it take to clean the toilet once this week?’) were also asked to provide more detail and granularity.

**Table 2:** Questions relating to servicing of CBS toilets and the SanQoL-5 index that participants were asked weekly, in addition to the possible responses and the value attributed to them

(a) Servicing questions Responses: Yes (1), No (0).	
(i)	Was there a problem with the collection service this week?* (If so, what problem? Collector came early (‘early’), collector did not come (‘not_come’), cover material not received (‘not_recieved_cover’), do not know, other)
(ii)	Was a clean cartridge available when you needed it?
(iii)	Was the replacement cartridge cleaned to your satisfaction?
(iv)	Was your CBS cartridge/bucket changed often enough?
(v)	Was the toilet cleaned to your satisfaction?
**(b) SanQoL-5 questions** Responses: always (3), sometimes (2), rarely (1), never (0), do not know	
(i)	Can you use your usual toilet without feeling disgusted?
(ii)	Can you use your usual toilet without worrying that it spreads diseases?
(iii)	Can you use your usual toilet in private, without being seen?
(iv)	Can you use your usual toilet without feeling ashamed for any reason?
(v)	Are you able to feel safe while using your usual toilet?

*For question (a(i)), the values assigned to responses are reversed due to the phrasing of the question, where a ‘no’ response is a positive sentiment and scored as 1 and a ‘yes’ response is negative and scored as 0.

Participants were also asked weekly about their sanitation-related quality of life (SanQoL-5 index) in the previous week using five questions, as defined by Ross et al. (2022) (Table 2b). The answers to each set of questions—servicing and SanQoL-5—were given numerical values (Table 2) that were summed to give an overall ‘score’, to evaluate the perceived SanQoL-5 (out of 15) in relation to CBS toilet servicing (out of 5); with ‘scores’ normalized to be a proportion of 1. An equal weighting was used on all SanQoL-5 questions as it had not yet been validated in any of Kenya, Peru or South Africa at the time of analysis. The SanQoL-5 index has since been validated in Kenya and does recommend altering weighting based on the values of the population in a given country^76,77^. Overall SanQoL-5 and servicing ‘scores’ were calculated for each weekly set of responses for each participant. The data were filtered to exclude weeks where not all servicing and SanQoL-5 questions were answered by that participant. Engagement percentage was calculated for each participant (A.C. et al., manuscript in preparation)—based on the number of valid weekly responses in the combined dataset from each participant, assuming 52 survey weeks.

Two approaches were adopted to evaluate SanQoL-5 metrics in relation to servicing of CBS toilets. First, individual weekly scores and secondly an average score per participant over the entire study as they provide unique insights to participant satisfaction.

### Software and statistics

Data analysis, visualization and statistical evaluations were conducted using a combination of Microsoft Excel and JMP Pro 17. Chi-squared tests were used to assess differences in reported problems between groups (nominal and ordinal data), with a significance threshold set at *P* = 0.05. Differences in user average SanQoL-5 and servicing scores were evaluated using Kruskal–Wallis with pairwise Wilcoxon post hoc tests (*P* = 0.05) as neither SanQoL-5 nor servicing scores were not normally distributed (Shapiro–Wilk test), thus failing the assumptions of analysis of variance (ANOVA).

## Supplementary information

Supplementary Information Supplementary Appendices A–C.

## Data Availability

The data are publicly available via the ReShare service (10.5255/UKDA-SN-857073): Longitudinal Sanitation Data From High-Frequency Phone Surveys Across Three Countries, 2020–2024.
