# Supplementary material for: Assessing the role of servicing in enhancing sanitation-related quality of life among container-based sanitation users
Source: Nat Water. 2025 Sep 25;3(10):1163–73. doi: 10.1038/s44221-025-00508-6 (PMC12537483; doi:10.1038/s44221-025-00508-6)
Supplement: Supplementary file 1 — Supplementary Appendices A–C. [file 44221_2025_508_MOESM1_ESM.pdf]

nature water

Supplementary information

https://doi.org/10.1038/s44221-025-00508-6

Assessing the role of servicing in enhancing sanitation-related quality of life among container-based sanitation users

In the format provided by the authors and unedited

Supplementary Information

Appendix A – Toilet problems and severity

Appendix A-1: The total number of responses to weekly questions on whether participants had a problem with their primary toilet that week, also broken down by country and primary toilet type. Percentages for "CBS-users" and "non-CBS" denotes the proportion of respondents who described their primary toilet as either CBS or non-CBS for each country.

	Total responses	CBS-users	Non-CBS
**Kenya**	3,838	1,931 (50.3%)	1,907 (49.7%)
**Peru**	1,678	919 (54.8%)	759 (45.2%)
**South Africa**	1,605	914 (56.9%)	691 (43.1%)
**ALL**	7,121	3,764 (52.9%)	3,357 (47.1%)

Appendix A-2: Distribution of participant numbers by country for questions on problems with toilets and the severity of problems.

		Kenya	Peru	South Africa	All
**Gender**	**Female**	64	86	60	210
	**Male**	44	10	38	92
**Age Group**	**18-19**	5	5	0	10
	**20-24**	31	8	10	49
	**25-29**	31	8	12	51
	**30-34**	17	13	10	40
	**35-39**	9	14	17	40
	**40-44**	5	19	16	40
	**45-49**	6	13	17	36
	**50-54**	2	11	8	21
	**55-59**	1	2	3	6
	**>60**	1	3	5	9
**Toilet Type**	**CBS**	55	45	48	148
	**Non-CBS**	53	51	50	154
Total	**-**	*108*	*96*	*98*	*302*

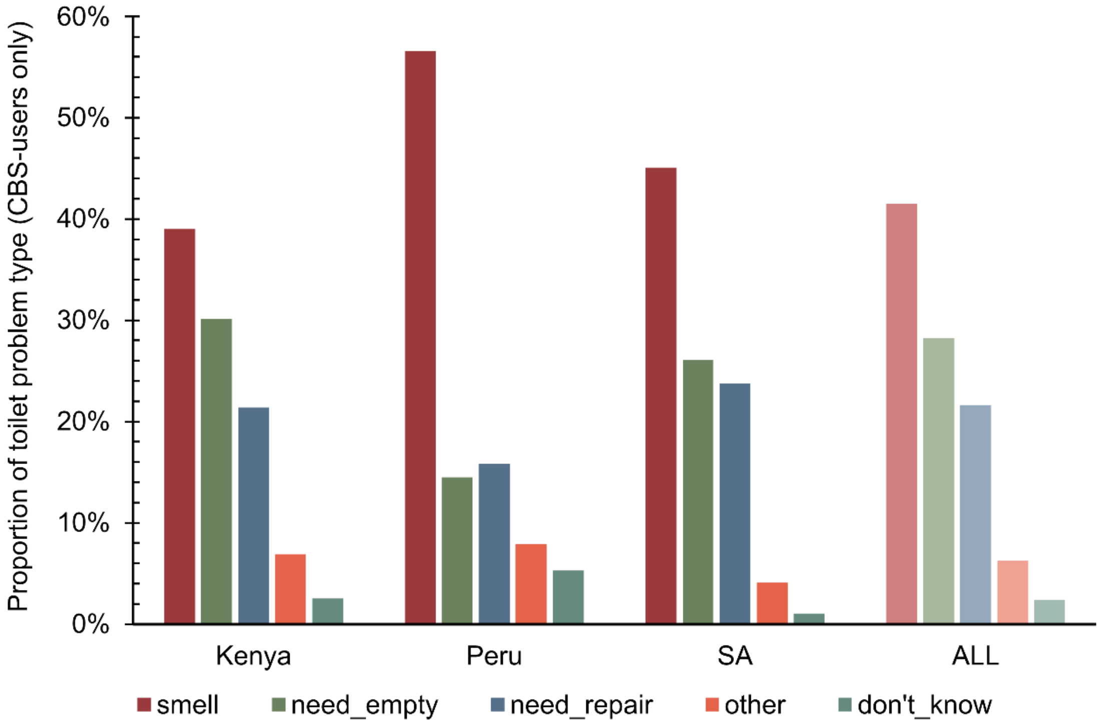

Appendix A-3: The specific problem types when CBS-users recorded that they had a problem with their toilet that week (and the severity of, Error! Reference source not found.**). Options included smelled bad ("smell", red), needs emptying ("need_empty", green), needs repairs ("need_repair", blue), other (orange), and don't know (teal).**

Appendix B – Servicing of CBS toilets

Appendix B-1: Distribution of participant numbers by country for CBS servicing questions.

		Kenya	Peru	South Africa	All
**Gender**	**Female**	32	31	41	104
	**Male**	27	9	26	62
**Age Group**	**18-19**	2	2	0	4
	**20-24**	15	3	8	26
	**25-29**	18	2	5	25
	**30-34**	7	6	6	19
	**35-39**	6	4	15	25
	**40-44**	3	11	8	22
	**45-49**	4	5	15	24
	**50-54**	2	5	4	11
	**55-59**	1	1	2	4
	**>60**	1	1	4	6
Total	**-**	*59*	*40*	*67*	*166*

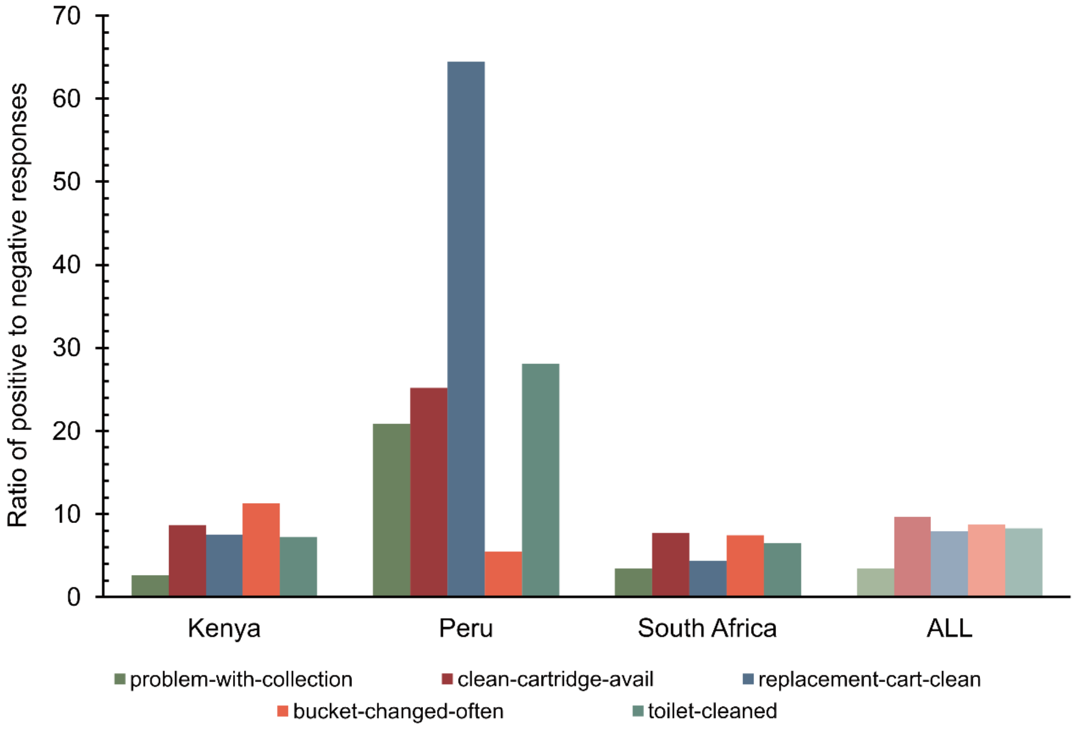

Appendix B-2: The ratio of positive to negative responses for CBS servicing questions, per country (Error! Reference source not found.**a). The ratio value denotes how many more times as many positive as negative responses for that question.**

Appendix C – The relationship between CBS servicing and sanitation-related quality of life

Appendix C-1: Distribution of participant numbers by country for CBS servicing and SanQoL-5 questions.

		Kenya	Peru	South Africa	All
Gender	Female	29	29	33	91
	Male	20	6	9	35
Age Group	18-19	1	2	0	3
	20-24	13	3	4	20
	25-29	14	2	2	18
	30-34	7	3	3	13
	35-39	5	3	12	20
	40-44	3	10	4	17
	45-49	2	5	10	17
	50-54	2	5	2	9
	55-59	1	1	2	4
	>60	1	1	3	5
Total	-	*49*	*35*	*42*	*126*

Appendix C-2: Results of fitting models to SanQoL-5 and servicing scores which demonstrate low positive corelation. Models reported have been fit to all participants, except for linear model which has also been done per country. Model fit (solid red line) and confidence interval curves (dashed red line with red shading fill) are plotted on Servicing score (x) against SanQoL-5 score (y) plots; jitter has been added to individual participant scores (random uniform, 0.05 limit).

Model	Country	Average participant scores	Individual participant scores
Linear	Overall	r^2 = 0.0765 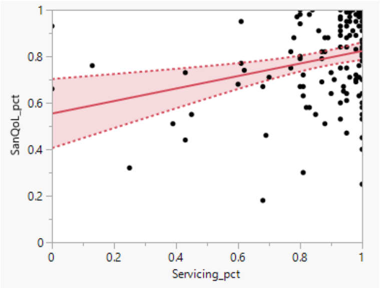	r^2 = 0.1453 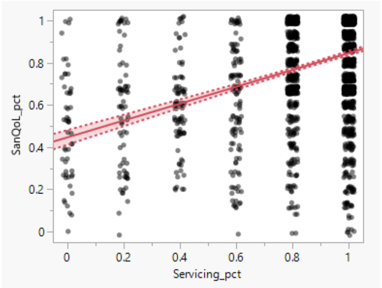

	Kenya	$r^2 = 0.3313$ 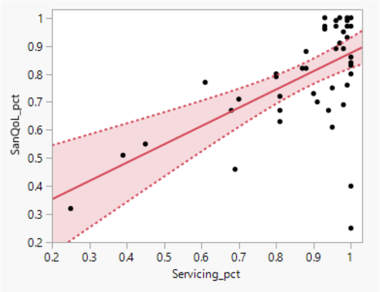	$r^2 = 0.2589$ 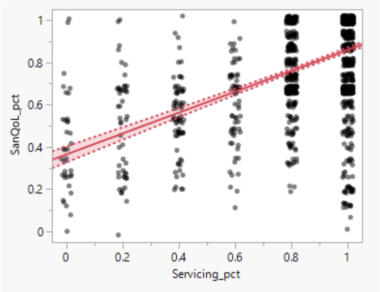
	South Africa	$r^2 = 0.0008$ 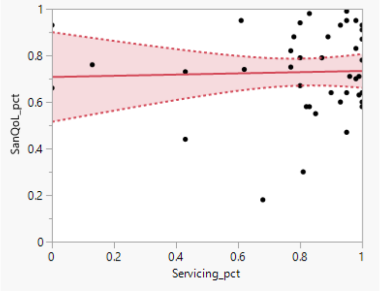	$r^2 < 0.0001$ 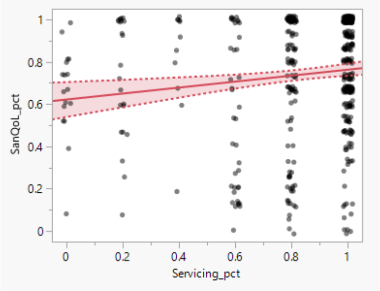
	Peru	$r^2 = 0.0014$ 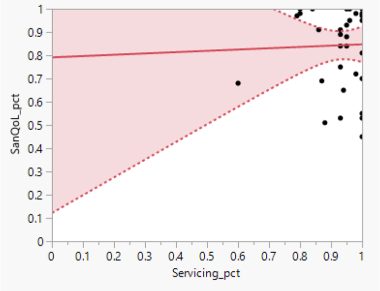	$r^2 = 0.0174$ 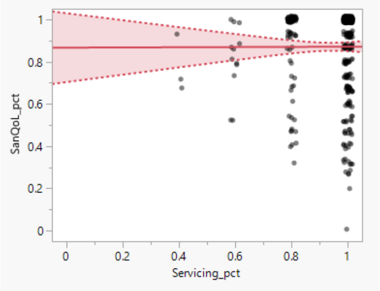
**Quadratic**	**Overall**	$r^2 = 0.1072$	$r^2 = 0.1482$
**Cubic**	**Overall**	$r^2 = 0.1297$	$r^2 = 0.1484$
**Quartic**	**Overall**	$r^2 = 0.1299$	$r^2 = 0.1521$

Appendix C-3: Mean SanQoL-5 and Servicing scores per country over time (points) with linear regression (lines, R^2 given) and confidence intervals (shaded area).

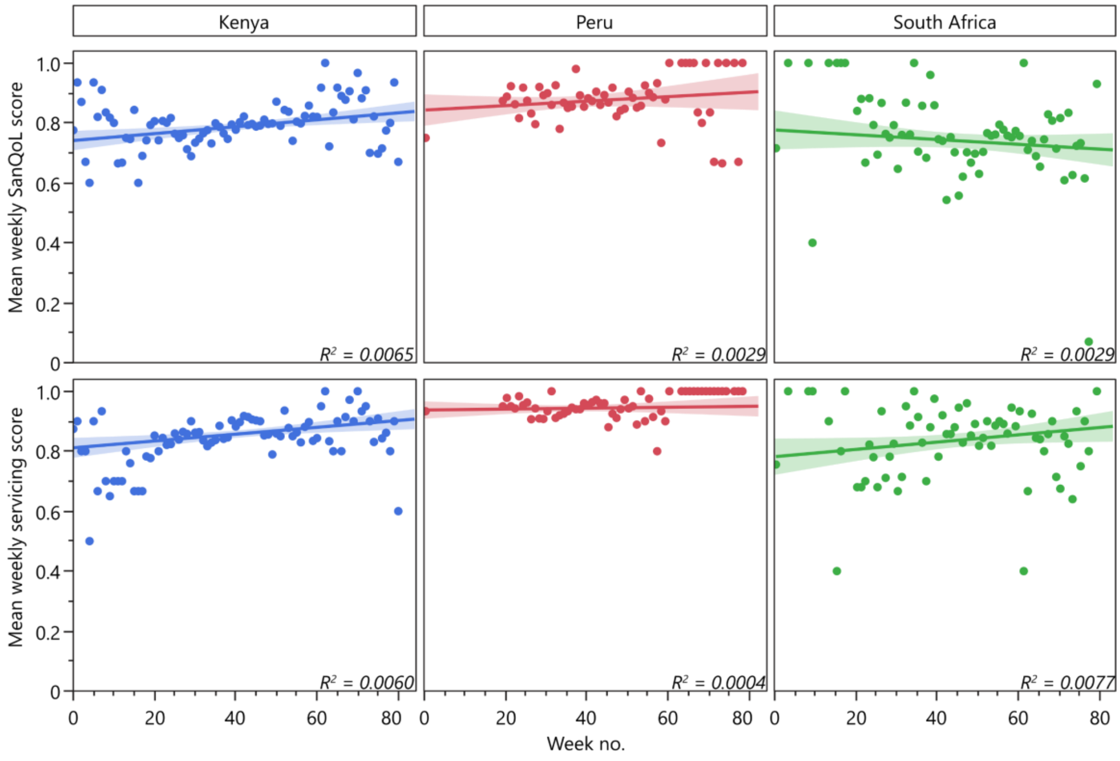

C.1. Valid responses and engagement percentage

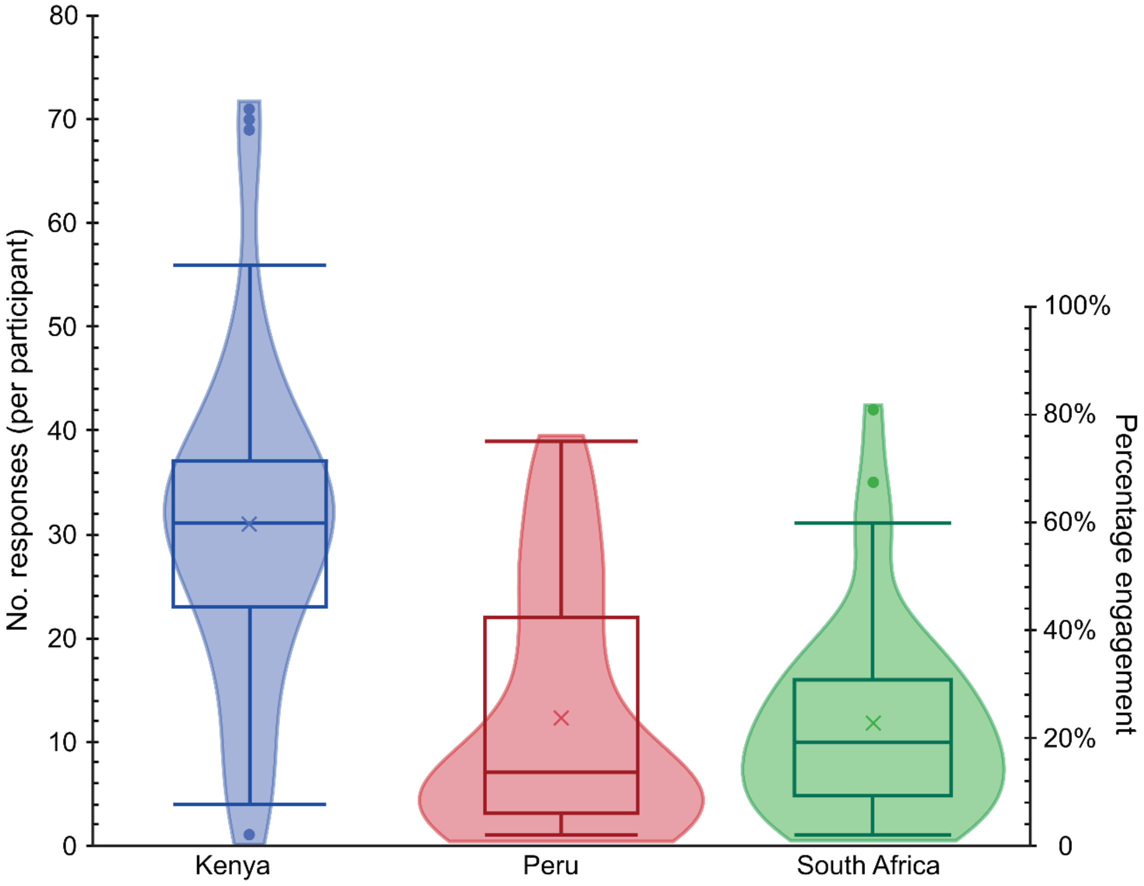

Appendix C-4: The spread of number of valid responses per participant for sanitation-related quality of life, servicing, and primary toilet questions per country. Kenya (blue), Peru (red), and South Africa (green). Violin plots show the density of responses, box plots show mean (cross), median (line), interquartile range (box), range up to 1.5x the interquarrtile range (whiskers), and outliers (dots). Second y-axis denotes percentage engagement (assuming max. 52-weeks), number of datapoints in boxplots for Kenya (49), Peru (35), and South Africa (42).

Appendix C-5: Engagement percentage summary statistics of each participant per country, based on 52 weeks possible contributions.

	Kenya	Peru	South Africa	(All)
**Mean**	59.5%	23.6%	22.6%	(37.2%)
**Std Dev**	30.2%	22.8%	18.4%	(30.4%)
**Std Err**	9.1%	5.2%	3.4%	(9.2%)
**Median**	59.6%	13.5%	19.2%	(30.8%)
**Interquartile range**	26.9%	36.5%	21.7%	(48.6%)
**Lower quartile**	44.3%	5.8%	9.1%	(11.0%)
**Upper quartile**	71.2%	42.3%	30.8%	(59.6%)
**Range**	134.6%	73.1%	78.9%	(134.6%)
**Min**	1.9%	1.9%	1.9%	(1.9%)
**Max**	136.5%	75.0%	80.8%	(136.5%)

C.2. SanQoL-5 and servicing summary statistics

Appendix C-6: Summary statistics of participant-average percentage scores for sanitation-related quality of life (SanQoL-5, teal) and toilet servicing (purple) questions that CBS-users were asked, by country.

	Kenya		Peru		South Africa	
	SanQoL-5	Servicing	SanQoL-5	Servicing	SanQoL-5	Servicing
**Mean**	0.80	0.89	0.84	0.93	0.73	0.81
**Std Dev**	0.19	0.17	0.17	0.09	0.18	0.26
**Variance**	0.04	0.03	0.03	0.01	0.03	0.07
**Median**	0.83	0.96	0.91	0.95	0.74	0.90
**Interquartile Range**	0.28	0.15	0.28	0.07	0.27	0.21
**Lower quartile**	0.70	0.84	0.70	0.93	0.62	0.78
**Upper quartile**	0.97	0.99	0.98	1.00	0.89	0.98
**Range**	0.75	0.75	0.55	0.40	0.81	1.00
**Min**	0.25	0.25	0.45	0.60	0.18	0.00
**Max**	1.00	1.00	1.00	1.00	0.99	1.00

Appendix C-7: Summary statistics of individual weekly scores per participant for sanitation-related quality of life (SanQoL-5, teal) and toilet servicing (purple) questions that CBS-users were asked, by country.

	Kenya		Peru		South Africa	
	SanQoL-5	Servicing	SanQoL-5	Servicing	SanQoL-5	Servicing
**Mean**	0.79	0.86	0.87	0.94	0.74	0.84
**Std Dev**	0.23	0.24	0.20	0.11	0.28	0.26
**Variance**	0.06	0.06	0.04	0.01	0.08	0.07
**Median**	0.87	1.00	1.00	1.00	0.80	1.00
**Interquartile Range**	0.33	0.20	0.20	0.00	0.40	0.20
**Lower quartile**	0.67	0.80	0.80	1.00	0.60	0.80
**Upper quartile**	1.00	1.00	1.00	1.00	1.00	1.00
**Range**	1.00	1.00	1.00	0.60	1.00	1.00
**Min**	0.00	0.00	0.00	.40	0.00	0.00
**Max**	1.00	1.00	1.00	1.00	1.00	1.00
